# A novel synthetic small molecule YH-306 suppresses colorectal tumour growth and metastasis *via*FAK pathway

**DOI:** 10.1111/jcmm.12450

**Published:** 2014-10-29

**Authors:** Fujun Dai, Yihua Chen, Li Huang, Jinhua Wang, Tao Zhang, Jingjie Li, Weiguang Tong, Mingyao Liu, Zhengfang Yi

**Affiliations:** a Shanghai Key Laboratory of Regulatory Biology, Institute of Biomedical Sciences and School of Life Sciences, East China Normal UniversityShanghai, China; b The Key Laboratory of Natural Medicine and Immuno-Engineering, Henan UniversityKaifeng, China; c Center for Cancer and Stem Cell Biology, Institute of Biosciences and Technology, Department of Molecular and Cellular Medicine, Texas A&M University Health Science CenterHouston, TX, USA

**Keywords:** small molecule, colorectal tumour, metastasis, FAK pathway

## Abstract

Cell migration and invasion are key processes in the metastasis of cancer, and suppression of these steps is a promising strategy for cancer therapeutics. The aim of this study was to explore small molecules for treating colorectal cancer (CRC) and to investigate their anti-metastatic mechanisms. In this study, six CRC cell lines were used. We showed that YH-306 significantly inhibited the migration and invasion of CRC cells in a dose-dependent manner. In addition, YH-306 inhibited cell adhesion and protrusion formation of HCT116 and HT-29 CRC cells. Moreover, YH-306 potently suppressed uninhibited proliferation in all six CRC cell lines tested and induced cell apoptosis in four cell lines. Furthermore, YH-306 inhibited CRC colonization *in vitro* and suppressed CRC growth in a xenograft mouse model, as well as hepatic/pulmonary metastasis *in vivo*. YH-306 suppressed the activation of focal adhesion kinase (FAK), c-Src, paxillin, and phosphatidylinositol 3-kinases (PI3K), Rac1 and the expression of matrix metalloproteases (MMP) 2 and MMP9. Meanwhile, YH-306 also inhibited actin-related protein (Arp2/3) complex-mediated actin polymerization. Taken together, YH-306 is a candidate drug in preventing growth and metastasis of CRC by modulating FAK signalling pathway.

## Introduction

Although screening, surgical resection and adjuvant therapy have improved the outcome for patients with colorectal cancer (CRC), CRC is still the third-leading cause of cancer-related deaths 1. More than 140,000 new cases of CRC were estimated and more than 50,000 individuals will die from CRC during 2013 in the United States 2. To address this conundrum, many studies focused on research for new therapies to target metastatic progression, as metastasis from the primary site of tumour growth to distant organs was the leading cause of cancer-related mortality 3.

Cancer metastasis involves a series of processes, including cell adhesion, spreading, migration, invasion and colonization in the new sites 4. These processes are regulated by a variety of proteins, including cellular adhesion molecules, chemokines and their receptors, MMPs, other metastasis proteins and their downstream signalling pathways 5. The adhesion of cells onto specific extracellular matrix (ECM) components induces the formation of focal adhesions, followed by the recruitment of cytoplasmic proteins, such as focal adhesion kinase (FAK), c-Src, cdc42, Rac, paxillin and talin 6. Among these molecules, the FAK/Src complex mediates signalling by binding to and phosphorylating downstream molecules. In response to extracellular stimuli, autophosphorylation of FAK on Tyr397 regulates focal adhesion disassembly through controlling the phosphorylation of paxillin on Tyr31 and Tyr118 7. Downstream of FAK signalling, the p85 subunit of PI3K binds to FAK resulting in PI3K phosphorylation, which leads to cell migration 8,9. The initiation of cell adhesion and focal adhesions is also associated with the small G-proteins Rho, cdc42 and Rac, which regulate actin cytoskeleton dynamics leading to the formation of cell protrusions 10. During cell migration, the actin-related protein 2/3 complex drives the polymerization of actin filaments to direct cell protrusion, influencing cell migration and invasion 11.

To explore potential drug candidates against CRC metastasis, we screened one type of novel aryl tetrahydro-β-carboline derivatives for their inhibition efficacy towards CRC cell phenotypic assays, and found that YH-306 showed a significant inhibitory effect on the progression and development of CRC. *In vitro*, YH-306 significantly inhibited CRC cell migration, invasion, proliferation and colonization, and induced CRC cell apoptosis. *In vivo*, YH-306 depressed CRC growth and suppressed hepatic/pulmonary metastasis. Finally, we found that YH-306 blocked the activation of FAK and FAK-related signalling pathway, leading to the suppression of CRC growth and metastasis.

## Materials and methods

### Reagents, cell and animals

The antibodies used in this study were anti-Arp3; C-myc, Bax, Bcl-xl, Cleaved Caspase3, FAK, Phos-FAK, Phos-PI3K, MMP2, MMP9, tissue inhibitor of metalloproteinases 1 (TIMP1), Paxillin, Phos-paxillin, Erk, Phos-Erk (Cell Signaling Technology), c-Src, Phos-c-Src (Santa Cruz Biotechnology) and Rac1 (Millipore). DAPI was purchased from Sigma-Aldrich. Phalloidin was provided by Invitrogen. All of the media were obtained from Gibco. All CRC cell lines were obtained from the American Type Culture Collection (ATCC). Medium was supplemented with 10% FBS, penicillin (100 units/ml) and streptomycin (100 units/ml). BALB/c mice and nude mice were from the National Rodent Laboratory Animal Resources, Shanghai Branch of China. All animal experimental protocols were approved by the Animal Investigation Committee of the Institute of Biomedical Sciences, East China Normal University.

### Chemical synthesis of compound YH-306

**Fig 1:**
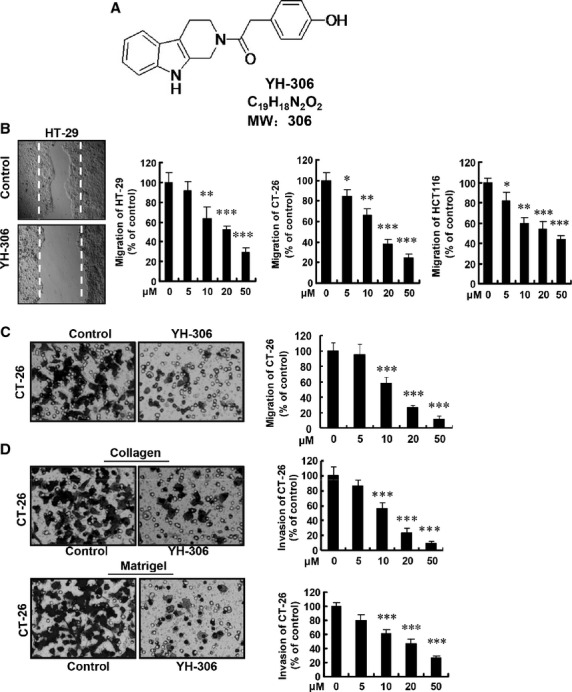
YH-306 suppresses the migration and invasion of colorectal cancer cells. (A) Chemical structure of YH-306 (molecular weight 306). (B) Inhibitory effect of YH-306 on wound healing migration of HT-29, CT-26 and HCT116 cells. Left panel, images of cell migration. Right panel, quantitative data for the relative percentage of migrated cells. (C) YH-306 suppressed transwell migration of CT-26 cells. (D) YH-306 inhibited the invasion of CT-26 cells through type I collagen (Upper panel) or Matrigel (Lower panel). *P < 0.05; **P < 0.01; ***P < 0.001.

As previous report 12,13, compound YH-306 (Fig.1A) was synthesized by our laboratory with greater than 98% purity. To a solution of 4-hydroxyphenylacetic acid (152 mg, 1.0 mmol) in anhydrous *N*,*N*-dimethylformamide (5.0 ml) was added 1-(3-dimethylaminopropyl)-3-ethyl-carbodiimide hydrochloride (249 mg, 1.3 mmol) and 1-hydroxy-benzotriazole (149 mg, 1.1 mmol) at 0°C, the reaction mixture was stirred for 10–15 min., then 1,3,4,9-tetrahydro-β-carboline (172 mg, 1.0 mmol) was added. The mixture was stirred for another 3 hrs at room temperature and diluted with H_2_O and extracted with ethyl acetate (3 × 60 ml). The combined organic phase was dried over anhydrous sodium sulphate, then concentrated in vacuo and the crude product was chromatographed over silica gel to give 199 mg (65% yield) of compound YH-306.

### Migration and invasion assays

For CRC cells, the migration and invasion assay was performed as previously reported 14. After overnight starvation, the cells were scratched with a pipette tip followed by the addition of YH-306. Images were taken after 16 hrs, and migrated cells were counted. The migration and invasion of CRC cells was assessed by the modified Boyden's chamber assay in 24-well cell culture plate with 8-μm pore.

### Cell adhesion and cell spreading assays

Adhesion and spreading assays were performed with some modifications 15. The 96-well plates were incubated with 2.5 μg fibronectin (Invitrogen) or 1 μg type I collagen. After treatment, tumour cells were added to each well and allowed to attach for 1 hr. Non-adherent cells were removed with PBS gently and adherent cells were stained with crystal violet. Spreading cells were identified as those cells that had distinguishable cytoplasm.

### Cell proliferation, live/dead and apoptosis assays

As previously reported 16, the effect of YH-306 on CRC cell proliferation was determined by MTS assay. LIVE/DEAD® Viability/Cytotoxicity kit (Invitrogen) was utilized to assess the cell death objectively by specified the protocol. Apoptosis of CRC cells induced by YH-306 was measured by annexin V-FITC/Propidium Iodide (PI, BD Biosciences) dual staining assay.

### Colony formation assay

The ability of isolated single cells to form colonies was tested using both two dimensional (2D) and 3D culture as described previously 17. All cells were stained with crystal violet. Experiments were set up in triplicate and medium was changed twice a week.

### Immunofluorescence

Immunofluorescence assays were performed according to the method previously described 18. The YH-306-treated cells were incubated with primary antibody overnight at 4°C, followed by incubation with secondary antibodies. A laser scanning confocal imaging system (Leica, Heidelberg, Germany) was used to observe stained cells.

### Animal models for tumour growth and metastasis

As previously reported 19, for tumour xenografts, 2 × 10^6^ HT-29 cells were injected into nude mice subcutaneously and the mice were treated consecutively for 20 days, with the measurement of bodyweight and the tumour dimensions. For metastasis, BALB/c mice were used. CT-26-luciferase (CT-26-luci) colon cancer cells were harvested and re-suspended. Hepatic metastases were generated by intrasplenic injection of 2.5 × 10^4^ of cancer cells and splenectomy. Pulmonary metastases were generated by tail vein injection of 2.5 × 10^4^ of cancer cells. Mice were killed after 2 weeks. All mice were intraperitoneally injected with YH-306 (20 and 50 mg/kg) every day, with an equivalent volume of dimethyl sulfoxide (DMSO) was injected in control animals (*n* = 5).

### Immunohistochemistry

Paraffin-embedded tissues were sectioned at 5 μm, haematoxylin and eosin staining was performed according to a standard protocol. Images were taken using a Leica microscope (DM4000B; Leica). The results were analysed with Image-Pro Plus 6.0 software.

### *In vitro* actin polymerization assay

This assay was performed as described 11 with some modifications. In brief, purified pyrene-labelled actin was re-suspended and incubated in general actin buffer for 1 hr on ice to depolyermize any actin oligomers, followed by micro-centrifugation at 4°C for 30 min. Exactly, 2 μM of actin alone or 2 μM of actin, 13 nM of Arp2/3 complexes and 100 nM of WASP protein VCA domain were incubated with DMSO (control) or 50 μM YH-306 for 15 min. on ice before pyrene actin fluorescence was measured over time.

### Western blot analysis

After the treatment of YH-306, cells were harvested and lysed in radio immunoprecipitation assay buffer containing protease/phosphotase inhibitors (Roche). Lysates were combined with sample loading buffer and heated at 100°C for 10 min. Protein samples were eluted in sample buffer and subjected to SDS-PAGE.

### Measurement of YH-306 binding to Arp2/3 using biolayer interferometry

Protein–small molecules interactions were examined with an Octet QK (FortéBio, Shanghai, China) by biolayer interferometry as described in previous studies 20–23. In brief, Arp2/3 protein complex was PEG-biotinylated with NHS-PEG4biotin (Thermo-Pierce), and buffer exchanged on PD-10 desalting columns. Then, biotinylated Arp2/3 protein complex was immobilized on streptavidin-coated fibre optic tips (FortéBio). YH-306 or CK-636, the positive control, was diluted into optimized binding buffer [25 mM Na HEPES (pH 8.0), 50 mM arginine-glutamate, and 150 mM NaCl].

### Statistical analysis

Results were statistically analysed using the Student's *t*-test with Microsoft Excel, and all experiments were repeated at least three times. A value of *P* < 0.05 was considered significant.

## Results

### YH-306 suppresses the migration and invasion of CRC cells

Migration is a key step during tumour metastasis, which is the critical reason for treatment failure and poor prognosis in patients with CRC 24. YH-306 was identified as a new inhibitor of CRC cell migration *via* screening more than 70 analogues. As shown in Figure1B, YH-306 significantly inhibited the migration of two human CRC cell lines (HCT116 and HT-29) and one mouse CRC cell line (CT-26) in a wound healing migration assay. To confirm the effect of YH-306 on migration, a transwell migration assay was performed and we found that migration of CT-26 cells was significantly reduced in a dose-dependent manner after treatment of YH-306, as shown in Figure1C. During metastasis, cancer cells need to pass through the basement membrane, and invade surrounding tissues to infiltrate distant organs 5. To assess the effect of YH-306 on this process, we used type I collagen and Matrigel as substrates. As shown in Figure1D, YH-306 evidently prevented CT-26 cells from invading the type I collagen- or Matrigel-coated membrane in a dose-dependent manner.

### YH-306 inhibits adhesion and spreading of CRC cells

**Fig 2:**
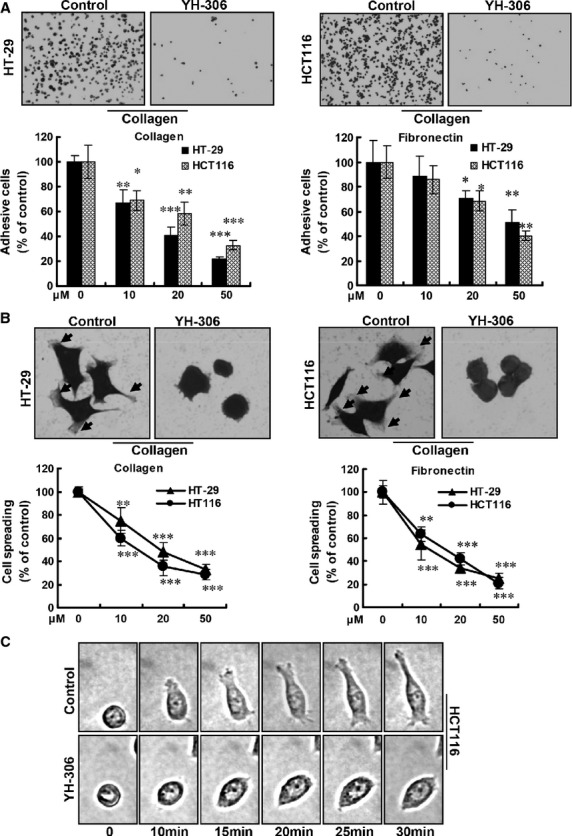
YH-306 inhibits cell adhesion and spreading of colorectal cancer cells. (A) Upper panel, representative images of cell adhesion. HT-29 or HCT116 cells were seeded onto type I collagen or fibronectin coated 96-well plates. Lower panel, results of cell adhesion are shown in the bar graph. (B) Upper panel, representative images of cell spreading. Lower panel, plot shows the per cent of cells that are spread for each treatment condition. (C) Time-lapse micrographs of representative cells exposed to 50 μM YH-306 or vehicle control. *P < 0.05; **P < 0.01; ***P < 0.001.

Cancer cell adhesion and cell spreading based on ECM components such as type I collagen or fibronectin are required for movement of metastatic cancer into new sites. Suppression of adhesion and spreading of CRC cells is therefore considered as a promising strategy for metastatic cancer therapy 15. To determine whether YH-306 inhibit CRC cell adhesion, we treated HCT116 and HT-29 seeded onto type I collagen or fibronectin with various concentrations of YH-306. As shown in Figure2A, 50 μM YH-306 significantly reduced HCT116 and HT-29 adhesion onto type I collagen or fibronectin. Quantitative data revealed that 50 μM YH-306 inhibited 67% of HCT116 cell and 78% of HT-29 cell attachment to type I collagen, and attachment to fibronectin was also significantly reduced by YH-306. These results showed that YH-306 significantly inhibited HCT116 and HT-29 cells attachment to type I collagen or fibronectin in a dose-dependent manner. Furthermore, we tested the effect of YH-306 on cell spreading, and results in Figure2B showed that YH-306 significantly suppressed cell spreading on type I collagen or fibronectin in a dose-dependent manner. Cells treated with YH-306 retained a rounded morphology (Fig.2B) and had defects in polarized extension (Fig.2C).

### YH-306 inhibits CRC cell growth and induces apoptosis

**Fig 3:**
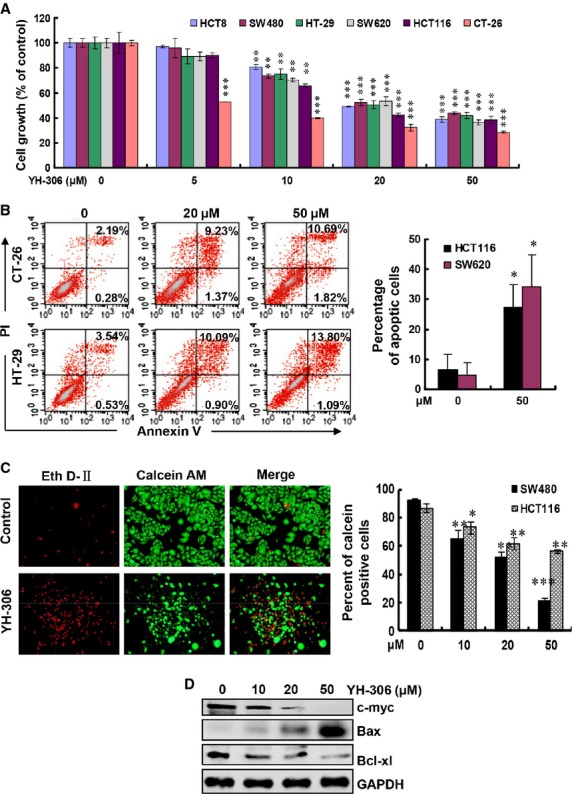
YH-306 inhibits colorectal cancer cell growth and induces apoptosis. (A) Effect of YH-306 on the proliferation of colorectal cancer cells was determined by MTS assay. The percentages of proliferating cells were relative to cells exposed to 0.1% DMSO. (B) Flow cytometry analysis was used to measure apoptosis of CT-26, HT-29, SW620 and HCT16 cells treated with YH-306 for 36 hrs. Cells positive for both propidium iodide (PI) and Annexin V were considered apoptotic. (C) Inhibitory effect of YH-306 on HCT116 and SW480 cells detected by Live&Dead assays. Left panel, representative graphs of SW480 cells were taken after treatment of 36 hrs. Right panel, quantification of the percentages of Calcein AM-positive cells. (D) Protein expression of c-myc, Bax and Bcl-xl in HT-29 cells treated by YH-306 or vehicle control. *P < 0.05; **P < 0.01; ***P < 0.001.

MTS assays were used to test the effect of YH-306 on the proliferation of CRC cells. As shown in Figure3A, YH-306 inhibited the growth of HCT8, HT-29, HCT116, SW480, SW620 and CT-26 cells in a dose-dependent manner after 48 hrs treatment. Fluorescence-activated cell sorting analyses in Figure3B revealed that 50 μM YH-306 increased apoptosis of HCT116, CT-26, HT-29 and SW620 cells by sevenfold, 5.2-fold, 3.6-fold and 3.4-fold respectively, compared with untreated cells. The percentage of live (Calcein AM positive) HCT116 cells decreased from 89.9% in control groups to 56.3% in 50 μM YH-306-treated groups and that decreased to only 23.3% in 50 μM YH-306-treated SW480 cells (Fig.3C). In addition, the percentage of Calcein AM-positive cells was reduced by YH-306 in a dose-dependent manner. Overexpression of c-myc is associated with the progression of CRC 25. YH-306 inhibited the expression of c-myc and Bcl-xl (Fig.3D), which is highly expressed in CRC and is correlated with prognosis of CRC 26. In contrast, YH-306 induced the expression of the pro-apoptotic protein Bax. These results suggested that YH-306 suppressed proliferation and induced apoptosis of CRC cells. Furthermore, to examine whether YH-306 also target other cancer cells, we carried out MTS assay to test the effect of YH-306 on prostate cancer cell LNCaP and breast cancer cell MDA-MB-231, and the results shown in Figure S1 displayed that YH-306 also inhibited cell growth of prostate cancer and breast cancer.

### YH-306 suppresses colony formation and xenograft tumour growth

**Fig 4:**
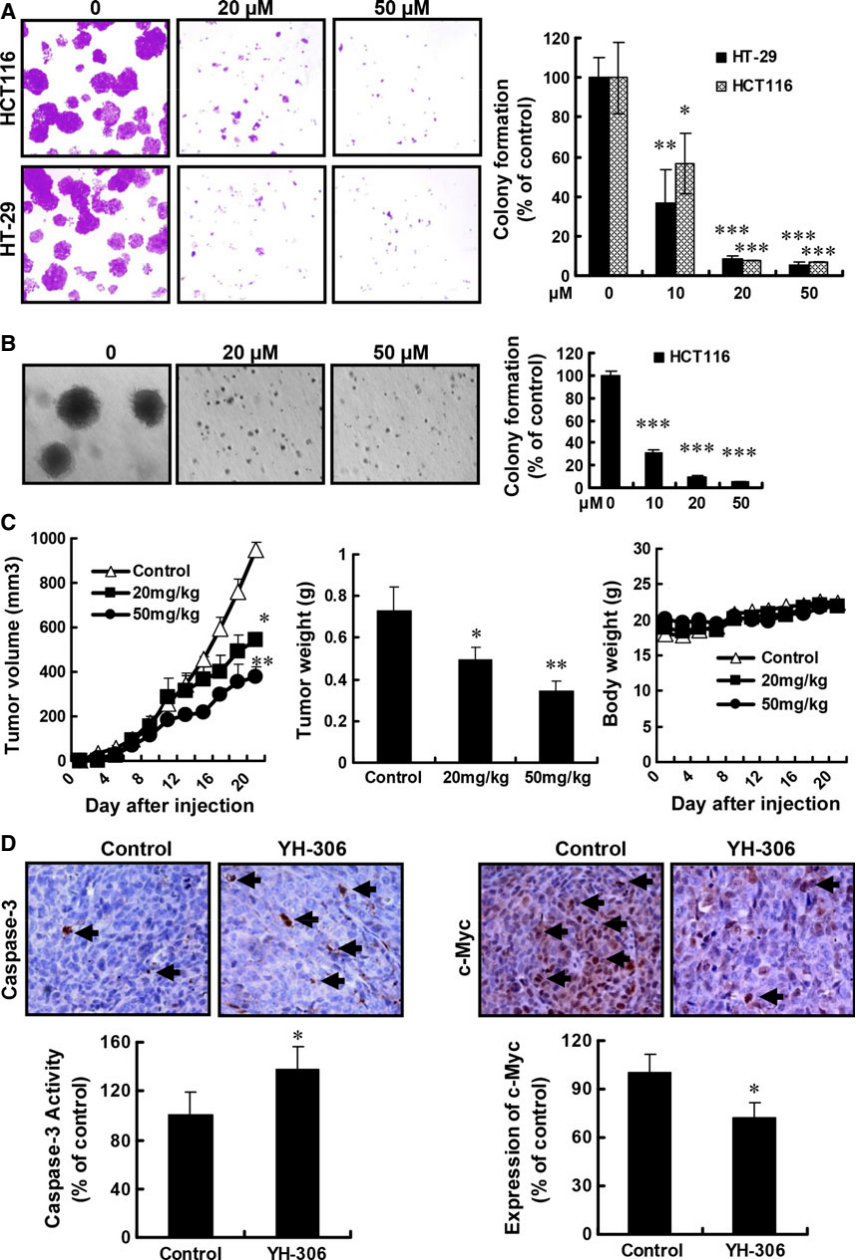
YH-306 suppresses colonization of colorectal cancer cell and inhibits xenograft colorectal cancer growth. (A) Suppression of 2D colony formation by various concentrations of YH-306 for 14 days. Images show representative cases at 14th day. Bar graph shows the relative number of HCT116 and HT-29 colonies. (B) In colonization assay, YH-306 inhibited the colony formation of HCT116 cells. The average colony formation from three independent experiments was shown. (C) After the injection of cancer cells, tumour volume and bodyweight were measured every 2 days. At the 20th day, the tumours were excised and tumour weight was recorded. (D) Immunohistochemistry analysis of cleaved caspase3 and c-myc expression in tumours from nude mice treated with DMSO or YH-306. Black arrows indicate the cells stained with specific antibody (upper panel). Quantitative data based on at least five independent sections from mice. *P < 0.05; **P < 0.01; ***P < 0.001.

The colony formation assay measures anchorage independent growth, and is considered the most stringent assay for mimicking the tumour growth *in vivo*. We first tested the effect of YH-306 on the 2D colony formation of HCT116 cells. As shown in Figure4A, the number of colonies in experimental groups treated by YH-306 at 10, 20 and 50 μM only was 56.7%, 7.5% and 6.9%, respectively, of the number of colonies formed by untreated groups. HT-29 cells generated results analogous to that of HCT116 cells. In 3D colony formation assays, YH-306 also inhibited colony formation of HCT116 cells significantly compared with the untreated controls (Fig.4B). Meanwhile, the size of the colonies formed in YH-306-treated groups is much smaller than that in untreated groups.

To test the effect of YH-306 on the growth of CRC *in vivo*, we performed tumour xenograft assays. After administration of 20 and 50 mg/kg/day YH-306 for 20 days, as shown in Figure4C, the average tumour volumes were 544.54 ± 32.15 mm^3^ (20 mg/kg/day) or 377.41 ± 44.13 mm^3^ (50 mg/kg/day), whereas that of untreated groups was 950.66 ± 34.30 mm^3^. In addition, the average tumour weight in untreated groups was 0.73 ± 0.11 g, and that in 20 or 50 mg/kg/day YH-306 groups was 0.50 ± 0.05 g or 0.34 ± 0.05 g respectively. However, the bodyweight of the mice treated with YH-306 had no obvious changes (Fig.4C). Furthermore, we noticed that the expression of c-myc was reduced in YH-306-treated groups (Fig.4D). In contrast, cleaved caspase3 expression was up-regulated in YH-306-treated groups (Fig.4D). All together, the above results implied that YH-306 suppressed primary tumour growth through inhibition of proliferation and induction of apoptosis.

### YH-306 reduces hepatic metastasis of CRC

**Fig 5:**
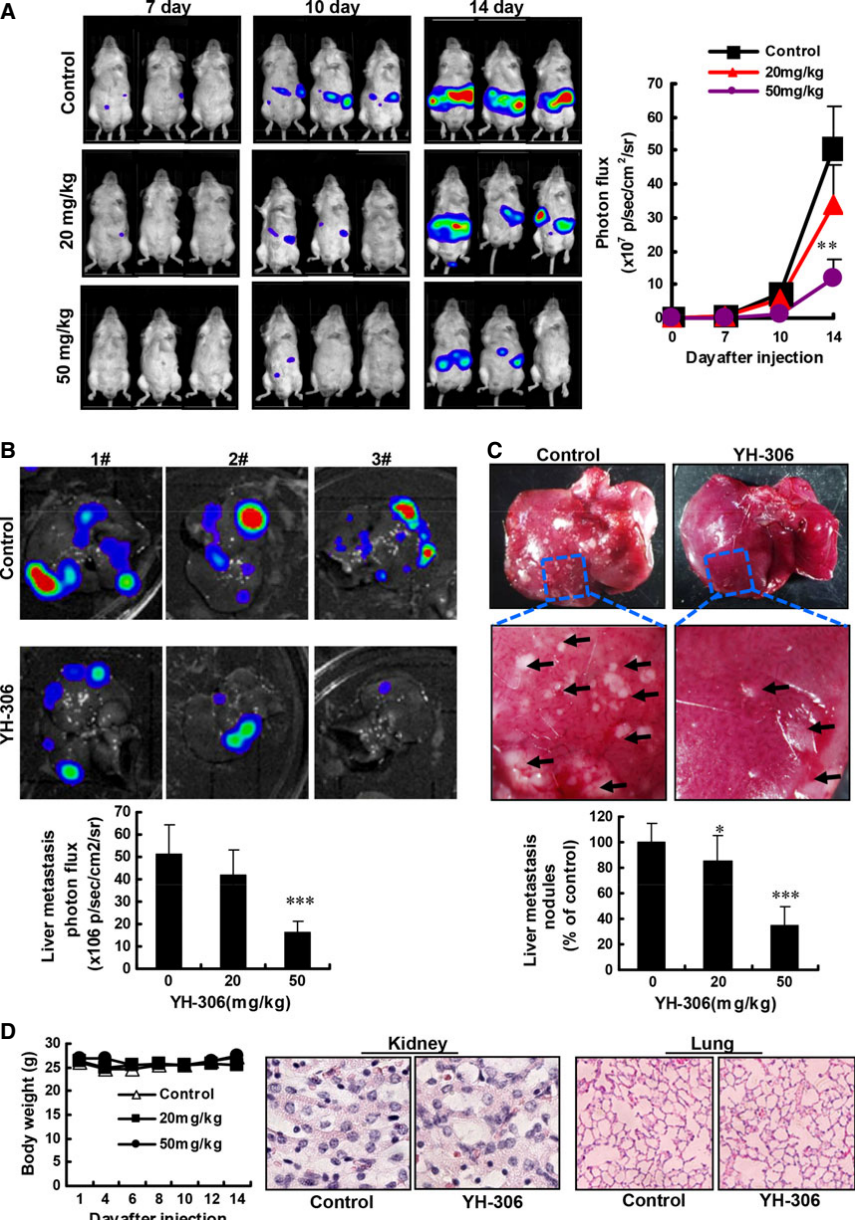
YH-306 reduces hepatic metastasis of colorectal cancer. (A) CT-26-luci cells were injected into the spleen of BALB/c mice. Representative images were taken at the 7th, 10th and 14th day respectively (left panel). Quantification of metastatic cells in whole body was determined by bioluminescence analysis (right panel). (B) Colorectal cancer cell metastases in liver and quantitative data of metastatic cells only in liver. (C) Upper panel, observational analyses of colorectal cancer metastases in livers from mice treated by YH-306 or DMSO. Lower panel, the cancer metastatic nodules on the liver surface were counted from mice. (D) The bodyweight of mice was measured. The kidney and lungs were excised from indicated mice, then fixed in 4% paraformaldehyde and stained with haematoxylin and eosin. *P < 0.05; **P < 0.01; ***P < 0.001.

Metastasis to distant organs is an ominous characteristic of cancer, and 80% of CRC patients with recurrent diseases develop hepatic metastasis 5. To examine YH-306 as a potential agent against hepatic metastasis, CT-26 cancer cells exogenously expressing luciferase (CT-26-luci) were injected into the spleen and mice were treated by two different dosages of YH-306 (20 and 50 mg/kg) or vehicle control every day. After treatment for 14 days, as shown in Figure5A, bioluminescence graphs showed that CT-26-luci cells largely localized in the abdomen region in untreated mice, and treatment of 50 mg/kg/day YH-306 significantly suppressed the metastasis with photon flux reduced by 77.02%. Then the mice were killed, and we found that tumour was not only localized in the liver but also spread to the intestines mesentery. To confirm the effect of YH-306 on hepatic metastasis, the liver was taken out and bioluminescence was examined. As shown in Figure5B, 50 mg/kg/day YH-306 treatment significantly suppressed hepatic metastasis with photon flux reduced by 67.60%. Furthermore, we counted the tumour nodules per liver, compared with the untreated group, and found that the number of tumour nodules in livers from mice treated by 20 and 50 mg/kg/day YH-306 was reduced by 14.39% and 65.31%, respectively (Fig.5C). Meanwhile, there was no obvious change in weight, or disruption in kidney or lung morphology in YH-306-treated mice (Fig.5D). These results suggested that YH306 inhibited hepatic metastasis of CRC *in vivo* with no significant side effects.

### YH-306 reduces pulmonary metastasis of CRC

**Fig 6:**
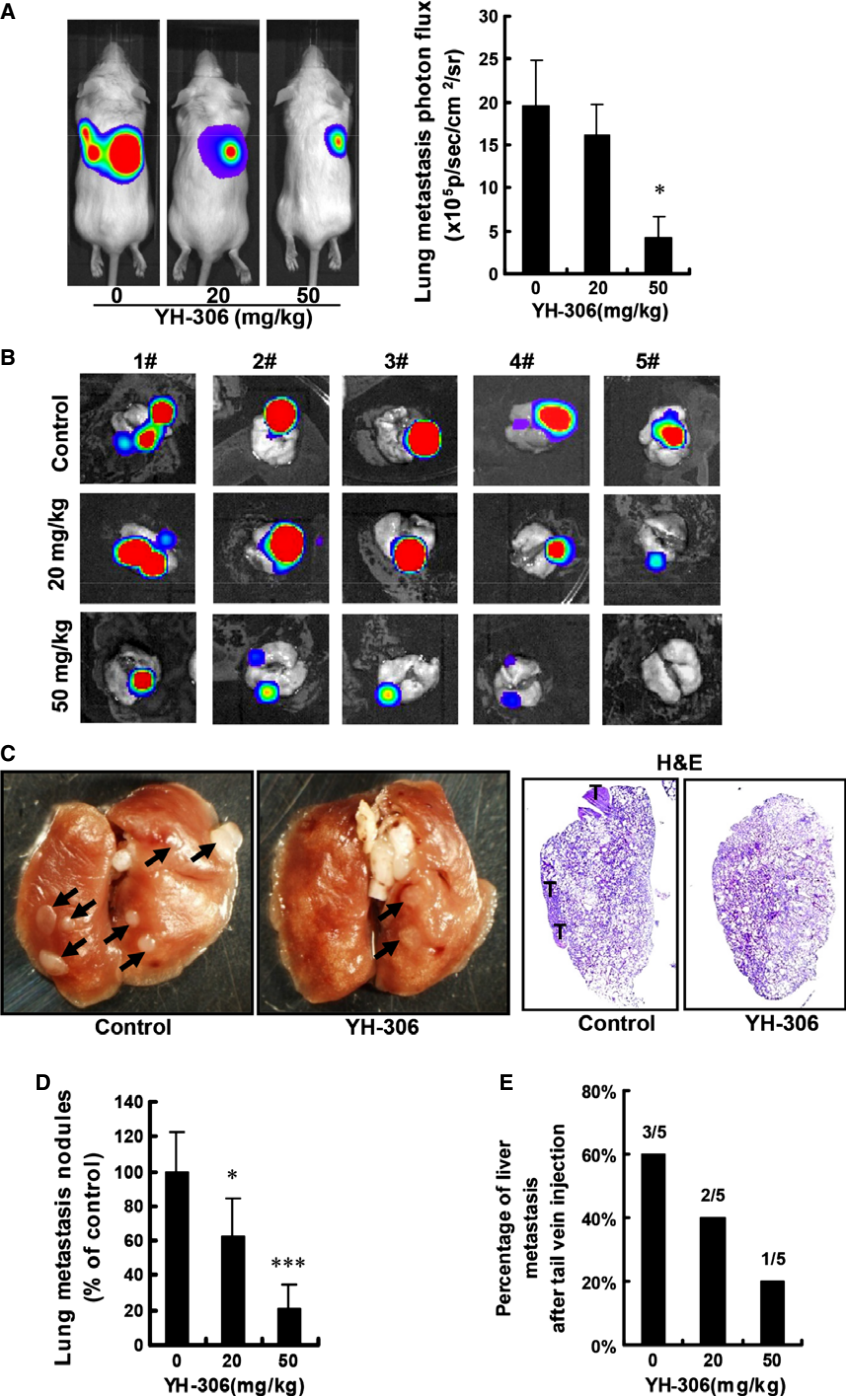
YH-306 reduces pulmonary metastasis of colorectal cancer. (A) After the administration of YH-306, representative images were taken at the 14th day (left panel) and quantification of metastatic cells in mice body was performed with bioluminescence analysis (right panel). (B) Mice were anaesthetized and lungs were removed from mice. Images show the metastases in lungs. (C) Left panel, observational analyses of colorectal cancer metastasis in lungs from mice treated with YH-306 or DMSO. Right panel, lungs from indicated mice were stained with haematoxylin and eosin. (D) The cancer metastasis nodules of lung surface from mice were counted. (E) The percentage of tail vein-injected mice with liver metastases in the colorectal cancer cell lung metastasis model. *P < 0.05; **P < 0.01; ***P < 0.001.

To evaluate the effect of YH-306 on pulmonary metastasis, which is one cause of fatality in patients with CRC 27, we used the pulmonary metastasis model based on tail vein injection. As shown in Figure6A and Figure6B, after treatment for 14 days, 20 mg/kg/day YH-306 showed no significant inhibitory effect on pulmonary metastases of CT-26-luci cells, 50 mg/kg/day YH-306 obviously inhibited pulmonary metastasis by 78.93%, compared with untreated controls. To confirm this result, the lungs were dissected from mice treated with YH-306 or vehicle control. YH-306 suppressed the number of tumour nodules in lung with significance (Fig.6C and D). We noticed that the liver is also a metastatic target organ after tail vein injection. As shown in Figure6E, colorectal tumour cells colonized the liver in 60% of untreated mice, whereas liver metastasis frequency was reduced to 40% and 20% respectively after administration of 20 and 50 mg/kg/day YH-306. These results indicated that YH-306 inhibited lung metastasis.

### YH-306 suppresses the activation of proteins in the FAK pathway

**Fig 7:**
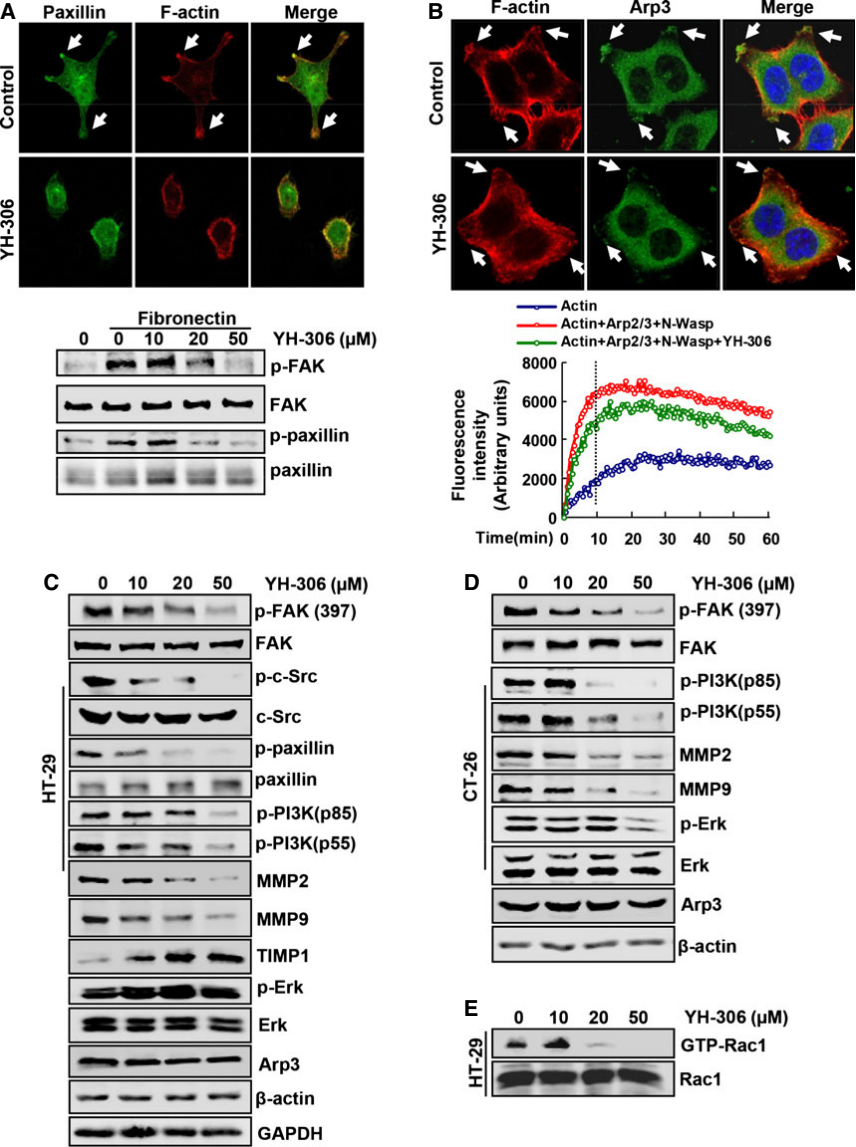
YH-306 suppresses FAK pathway and disrupts the recruitment of Arp3. (A) Focal adhesion formation in HT-29 cells treated with 0.1% DMSO or 50 μM YH-306 (upper panels). Phosphorylation of FAK and paxillin in HT-29 cells exposed to fibronectin and treated with YH-306 for 1 hr (lower panel). (B) Immunofluorescence of Arp3 in DMSO- or YH-306-treated HT-29 cells (upper panels) and time course of actin polymerization in the presence of 50 μM YH-306 (lower graph). (C and D) Colorectal cancer HT-29 (C) or CT-26 cells (D) were treated with YH-306, and whole-cell lysates were subjected to immunoblotting with antibodies against Arp3, Paxillin, PI3K, FAK, MMP9, MMP2, TIMP1 and Erk. (E) The activation of Rac1 was detected by pull down assays in HT-29 cells.

Focal adhesion formation plays an important role in cell motility 28. To understand the mechanisms involved in the suppressive effect of YH-306 on cell motility, we detected the effect of YH-306 on the distribution of F-actin and paxillin which are major components of focal adhesion. As shown in Figure7A upper panel, F-actin and paxillin were mainly localized in extended cell protrusions initially contacting with ECM. In contrast, after YH-306 treatment, cells retained a round morphology without the formation of protrusions and paxillin were distributed dispersedly, which is consistent with the results above (Fig.2C). FAK is a critical mediator in regulating cell motility including cell adhesion, spreading, migration and invasion, we found that the phosphorylation of FAK and paxillin was significantly reduced by YH-306 for 60 min. in cells exposed to fibronectin (Fig.7A).

To further clarify the mechanism that underlies the inhibitory effect of YH-306 on CRC cells, we measured activation of several proteins involved in FAK pathway using Western blot. As shown in Figure7C and D, after HT-29 and CT-26 cells were exposed to 50 μM YH-306 for 24 hrs, YH-306 significantly reduced the phosphorylation of FAK at Tyr397. We also performed experiments to determine whether YH-306 could act a direct inhibitor of FAK kinase activity *in vitro*, and found that YH-306 did not show significant inhibitory effect on FAK kinase activity *in vitro* compared to staurosporine as shown in Figure S2A and B. In HT-29 cells, as shown in Figure7C, the phosphorylation of proteins involved in the FAK pathway, including c-Src, paxillin and PI3K, was significantly reduced by 50 μM YH-306. The decreased phosphorylation of PI3K was also found in CT-26 cells treated by 50 μM YH-306 (Fig.7D). Activation of Rac1 is a vital component in FAK pathway regulating cell motility 29, and pull down assays revealed that YH-306 inhibited the activation of Rac1 GTPase in HT-29 cells (Fig.7E). To examine the individual importance of Src, PI3K and Rac1 for survival, adhesion and migration of CRC cell, we used Src inhibitor PP1, PI3K inhibitor LY294002 and Rac1 inhibitor NSC23766. The data shown in Figure S3 suggested that YH-306 suppressed cell growth, adhesion and migration of CRC cell through Src, PI3K and Rac1, but not depending on them.

It is reported that MMPs play an important role in the cancer metastatic cascade 30, and the expression of MMP2 and MMP9 is induced by FAK 31. In this study, we found that 50 μM YH-306 strongly reduced MMP2 and MMP9 expression in HT-29 cells. To confirm this effect of YH-306, we then measured the expression of TIMP1 and found that YH-306 up-regulated the expression of TIMP1 in a dose-dependent manner (Fig.7C). Meanwhile, in CT-26 cells, 50 μM YH-306 also showed significantly inhibitory effect on the expression of MMP2 and MMP9 as well as the phosphorylation of Erk (Fig.7D).

### YH-306 suppresses the distribution of Arp3 and inhibits Arp2/3 complex-mediated actin polymerization

The Arp2/3 complex drives many types of cell movement such as protrusion formation, cell spreading and actin polymerization 11,32. To examine the effect of YH-306 on the Arp2/3 complex, we examined the recruitment of Arp3. The recruitment of Arp3 to the protrusions of cells treated with YH-306 was reduced compared with untreated cells (Fig.7B). The cell morphology in Figure7A and B showed some defects in the cytoskeleton of cells treated with YH-306. In HT-29 and CT-26 cells, the expression of Arp3 did not show a significant change (Fig.7C and D), suggesting that YH306 inhibited the recruitment but not the expression of Arp3. To confirm whether this morphology result from the inhibition of Arp2/3 complex-regulated actin polymerization which is required for dynamic morphological changes 33, we performed Arp2/3 complex-regulated actin polymerization assays *in vitro*. As shown in Figure7B lower panel, YH-306 inhibited Arp2/3 complex-regulated actin polymerization at 10 min. 11. To further determine whether YH-306 could acts an ATP analogue and disturb ATP-dependent actin polymerization on its own, we perform ATP-dependent actin polymerization assay in which Arp2/3 complex and N-Wasp protein were not included. The result was shown in Figure S2C, suggesting that YH-306 did not show significant inhibitory effect on ATP-dependent actin polymerization at 10 min.

## Discussion

In this study, we demonstrated that a novel synthetic small molecule, YH-306, inhibited colorectal tumour growth and hepatic/pulmonary metastasis. The inhibitory effect of YH-306 resulted from the suppression of proliferation and motility. The potential mechanism by which YH-306 affected CRC may be because of inhibition of FAK and Arp2/3 pathway.

Liver and/or lungs are the typical sites of CRC metastases and hepatic/pulmonary metastasis account for the lethality in patients with CRC 5,27,34. So, effective anti-metastasis drugs for malignant CRC treatment are urgently needed. YH-306 prevented CRC cells from invading the liver after intrasplenic injection. Besides in the liver, colorectal tumours were also observed in the intestines mesentery (data not shown). Our data suggested that YH-306 also inhibited the localization of CRC to the intestines mesentery. Compared with the liver, the lungs are the second most frequently target organ of CRC 5. YH-306 inhibited lung metastasis of CRC cells, and suppressed the liver metastasis caused by tail vein injection of CRC cells (Figs5 and 6). In addition to the inhibition of CRC metastasis, YH-306 also inhibited colorectal tumour growth by inhibiting CRC proliferation and inducing cell apoptosis (Figs3 and 4). These results suggested that YH-306 inhibited the progression of CRC *in vivo*.

Migration is necessary for malignant cancer cells to invade adjacent tissues and metastasize to distant organs 35, and suppressing the initiation of migration is a promising approach to inhibit cancer cell dissemination 36. The process of migration underlies the invasion of cancer cells, and intravital microscopy studies displayed that cancer cells switch invasion strategies in response to ECM resulting in intravasation into blood vessels 37. YH-306 showed a significantly inhibitory effect on the migration and invasion of CRC cells in a dose-dependent manner (Fig.1). We also found that YH-306 was more effective at inhibiting migration and invasion than proliferation in CRC cells under the same concentrations and time (data not shown).

The process by which cancer cells attach to the vessel wall in distant organs and migrate out of the vessel is considered another crucial step of cancer metastasis 38. During this process, to interact with neighbouring cells and ECM, cell–cell and cell–ECM adhesions are formed 39, and cell protrusion and polarity is also important for invasive tumours 6,40. After YH-306 treatment, CRC cells HCT116 and HT-29 were prevented from attachment, and remained rounded without polarity as well as failed to form protrusions at the leading edge in response to type I collagen or fibronection stimuli (Fig.2). Consistent with these results, YH-306 treatment resulted in the reduction in filament-based cell–ECM contacts localized at cell adhesions and a random distribution of paxillin at the peripheral sites (Fig.7A, upper panel). These data implied that YH-306 inhibited migration and invasion resulting from interfering with initial processes in the metastatic cascade, which leads to the suppression of metastasis.

Cell migration is a highly integrated multistage process that is initiated by the protrusion of the cell membrane and focal adhesions 41,42. FAK and Arp2/3 are important factors during cell migration. FAK is correlated with the prognosis and clinicopathological parameters of some human malignancies, including CRC 43,44. Arp2/3 controls network of actin filaments and drives membrane protrusion during migration. Importantly, FAK and Arp2/3 complex associate and colocalize at transient structures formed early after adhesion during the cell migration 11. On the other hand, FAK/PI3K/Rac1 signalling also plays an important role in several kinds of cancer including breast cancer and CRC 45,46. Localization of FAK into adhesions induces its phosphorylation at Tyr397 and subsequently phosphorylation of c-Src and paxillin which also play an important role in focal adhesions 10,47,48. Phosphorylated PI3K induces the activation of Rac1 49. Rac1 activity induces the recruitment of Arp2/3 complexes 42, which is considered as a potential risk factor for liver metastasis of CRC 50,51. FAK also induces the expression of MMP2 and MMP9 accounting for cancer invasion and metastasis *via* ERK or JNK pathways 31,52,53. The effect of YH-306 on the ERK phosphorylation was weak, implying that YH-306 inhibited MMP2 and MMP9 expression *via* JNK pathway probably. In this study, YH-306 inhibited FAK/PI3K/Rac1 pathway activation and the recruitment of Arp3 as well as the expression of MMP2 and MMP9, resulting in the suppression of CRC metastasis. In addition, as shown in Figure7B, YH-306 suppressed the Arp2/3 complex-mediated actin polymerization which plays an important role in cell migration and invasion. Unlike the small molecule, CK636, used as a positive control compound, directly bound to Arp2/3 to block movement of Arp2 and Arp3 into their active conformation in the polymerization of actin (data not shown), which was consistent with previous reports 32,54. YH-306 did not bind to the Arp2/3 complex directly (data not shown), suggesting that YH-306 inhibited Arp2/3 complex-regulated actin polymerization *via* other means.

Collectively, in this study, we provided evidence that YH-306 inhibited CRC cell migration, invasion and proliferation *in vitro* resulting in the suppression of growth and metastasis *in vivo via* inhibition of the FAK pathways, implying that YH-306 might be valuable in preventing CRC growth and metastasis.

## Supporting Information

**Figure S1** YH-306 inhibits cell growth of prostate cancer and breast cancer.

**Figure S2** The effect of YH-306 on the FAK kinase activity *in vitro* and on the ATP-mediated actin polymerization.

**Figure S3** The effects of inhibitors on survival, adhesion and migration of colorectal cancer cell with or without YH-306.
