# Supplementary figures and images for: A novel synthetic small molecule YH-306 suppresses colorectal tumour growth and metastasis viaFAK pathway
Source: J Cell Mol Med. 2014 Oct 29;19(2):383–95. doi: 10.1111/jcmm.12450 (PMC4407606; doi:10.1111/jcmm.12450)

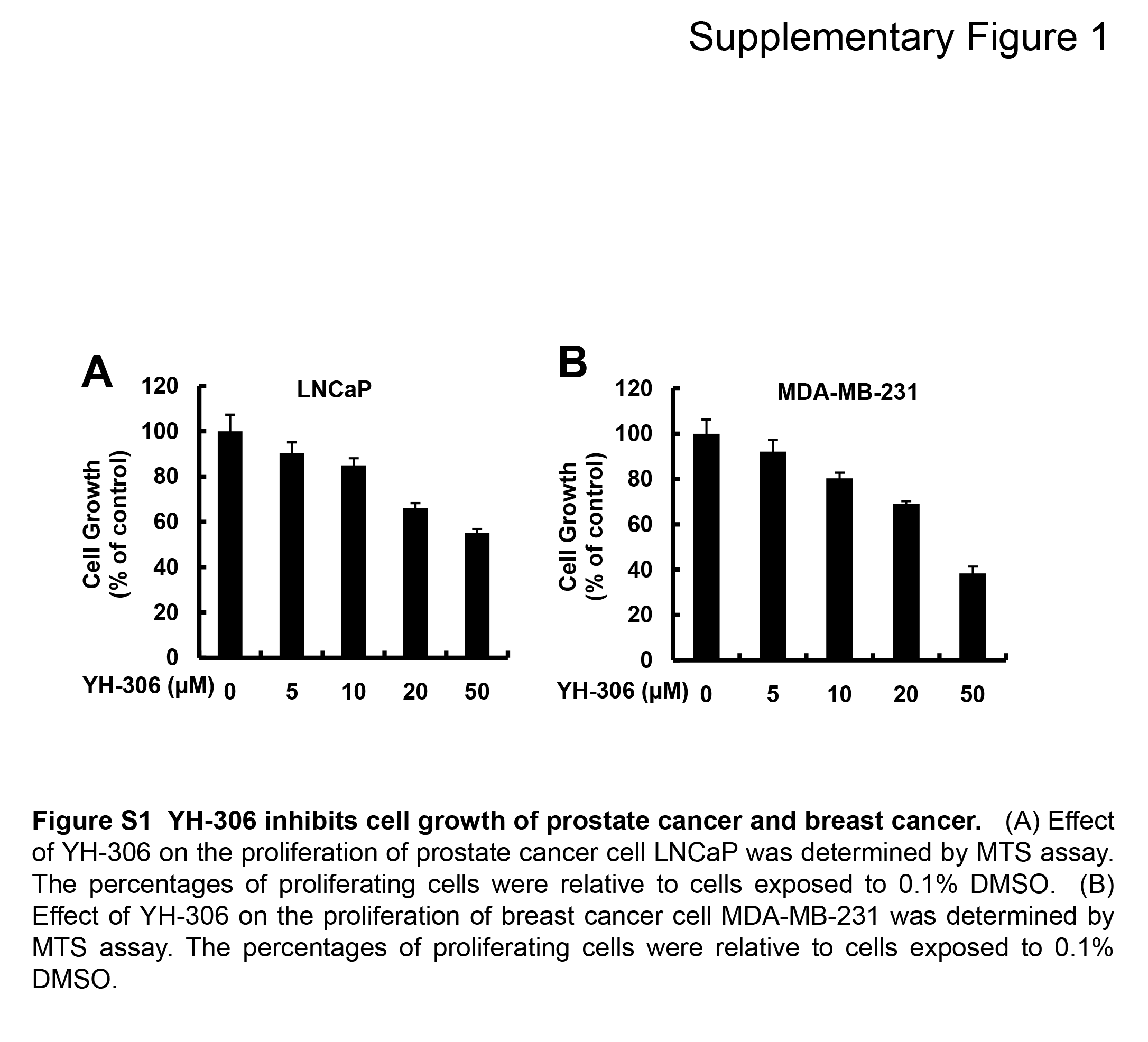

Supplement: Supplementary file 1 [file jcmm0019-0383-sd1.tif]

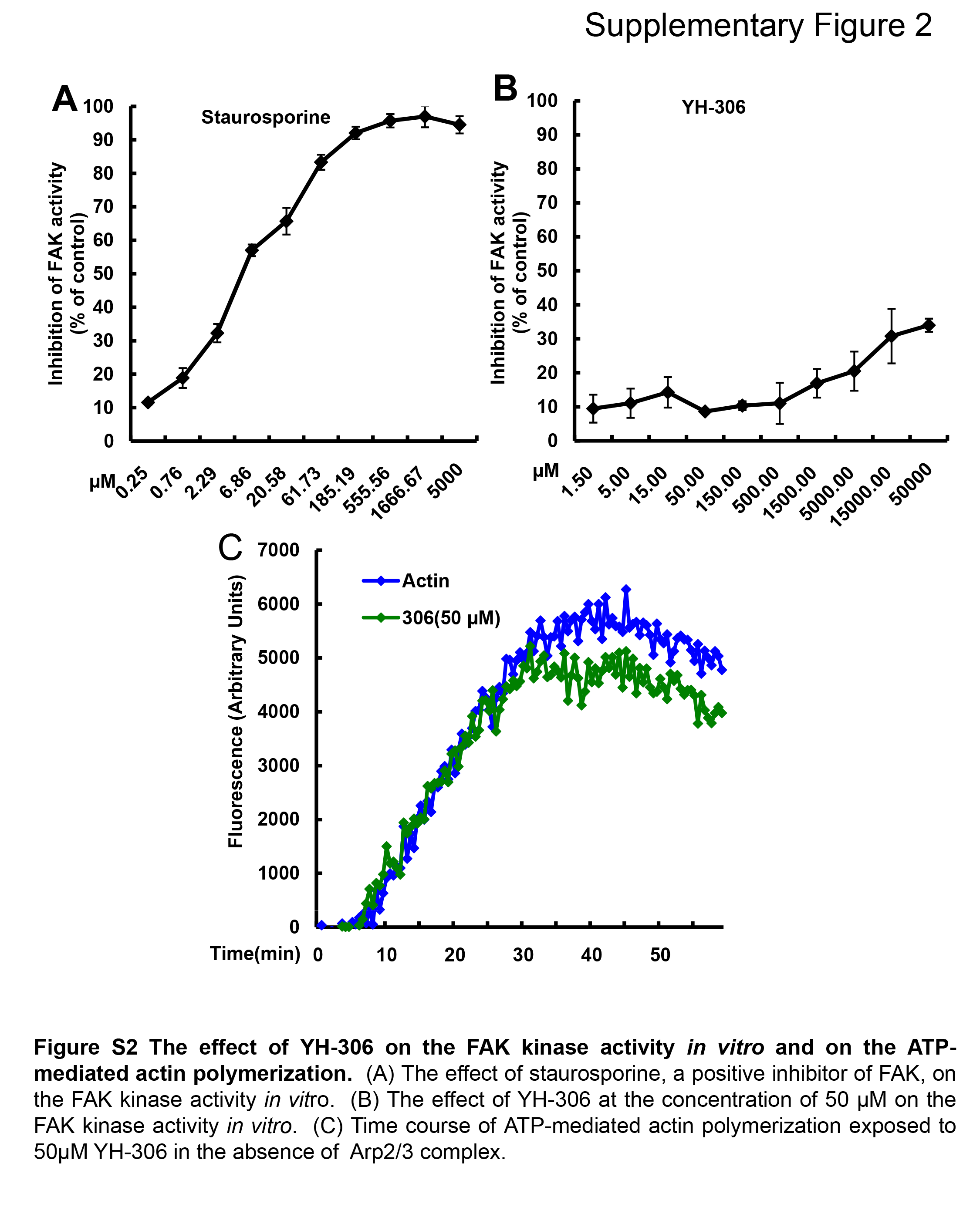

Supplement: Supplementary file 2 [file jcmm0019-0383-sd2.tif]

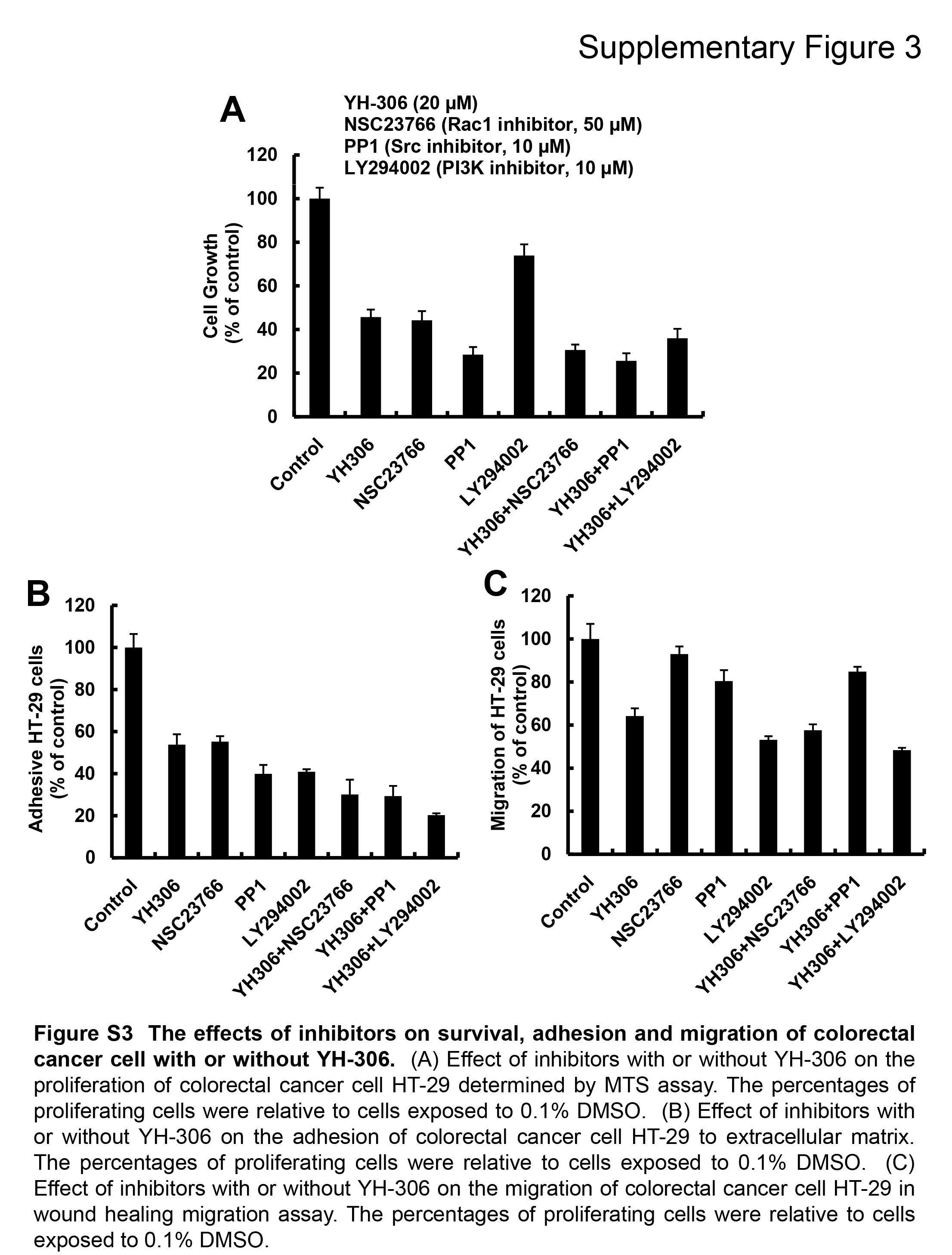

Supplement: Supplementary file 3 [file jcmm0019-0383-sd3.tif]
